# P-729. Prognostic Value of the Immunodeficiency Scoring Index for Respiratory Viral Infections in Hematopoietic Cell Transplant Recipients

**DOI:** 10.1093/ofid/ofae631.925

**Published:** 2025-01-29

**Authors:** Tali Shafat, Fareed Khawaja, Georgios Angelidakis, Layale Yaghi, Amy Spallone, Ella Ariza Heredia, Roy F Chemaly

**Affiliations:** The University of Texas MD Anderson Cancer Center, Houston, Texas; The University of Texas MD Anderson Cancer Center, Houston, Texas; The University of Texas Md Anderson Cancer Center, Houston, Texas; UT MD Anderson Cancer CEnter, Houston, Texas; University of Texas MD Anderson Cancer Center, Houston, Texas; The University of Texas MD Anderson Cancer Center, Houston, Texas; University of Texas MD Anderson Cancer Center, Houston, Texas

## Abstract

**Background:**

Accurate prediction of the severity of respiratory viral infections (RVIs) may help in improving patients’ outcomes. The respiratory syncytial virus (RSV) immunodeficiency scoring index (ISI), which incorporates patients’ host factors, was predictive of progression to lower respiratory tract infection (LRI) or mortality in HCT recipients with RSV infections. Our study goal was to correlate the ISI with severe respiratory infection and 30-day mortality in HCT recipients infected with different respiratory viruses and with non-viral respiratory infections.

**Table 1:**
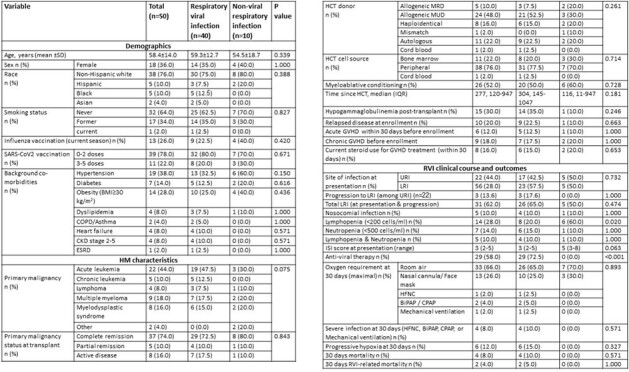
Baseline characteristics and clinical outcomes following respiratory infection. Abbreviations: BiPAP= bilevel positive airway pressure, BMI= Body mass index, CKD= chronic kidney disease, COPD= chronic obstructive pulmonary disease, CPAP= continuous positive airway pressure, ESRD= end-stage renal disease, GVHD= graft-versus-host disease, HCT= hematopoietic stem cell transplantation, HFNC= high-flow nasal cannula, ISI= immunodeficiency scoring index, IQR= interquartile range, LRI= lower respiratory tract infection, MRD= matched related donor, MUD= matched unrelated donor, RVI= respiratory virus infection, SD=standard deviation, URI= upper respiratory tract infection.

**Methods:**

In this prospective study, we enrolled 50 patients who had undergone allogeneic or autologous HCT and were infected with RSV (n=10), influenza (n=10), parainfluenza (n=10), SARS-CoV-2 (n=10) or had a non-viral respiratory infection (n=10). We calculated the ISI at enrollment and followed the patients for 4 weeks. Primary outcomes of interest were progression to LRI, the severity of RVI (the use of a high-flow nasal cannula, BiPAP, CPAP, or mechanical ventilation), and 30-day all-cause mortality.

**Figure 1:**
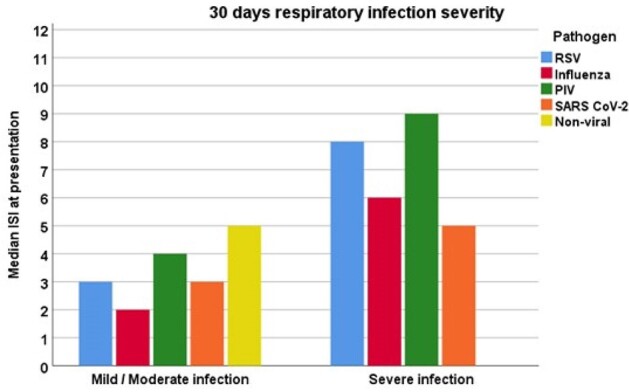
Median immunodeficiency scoring index at presentation and severity of respiratory infection (n=50). *Mild-moderate infection is defined as the maximal O2 requirement of a nasal cannula or face mask, and severe infection as the need for high flow nasal cannula, BiPAP, CPAP, or mechanical ventilation. Abbreviations: BiPAP= bilevel positive airway pressure, CPAP= continuous positive airway pressure, ISI= immunodeficiency scoring index, PIV= parainfluenza virus, RSV= respiratory syncytial virus, RVI= respiratory virus infection.

**Results:**

Among the 50 enrolled patients, the majority (78%) had undergone allogeneic HCT, while 22% had autologous HCT. Upon presentation, 44% were diagnosed with upper respiratory tract infection (URI), and 56% with LRI. Over the 30-day follow-up period, three patients with URI (14%) progressed to LRI, and four patients (8%) experienced severe infections leading to mortality (Table 1). For any viral pathogen, we observed higher ISI in patients with severe infections; in the non-viral respiratory infection group, relatively high ISI was noted at presentation but with no cases of severe infections (Figure 1). A higher ISI in HCT recipients with RVI was associated with 30-day mortality risk (median of 7 [IQR 5-8] vs. 3 [IQR 2-4], p=0.003) and severe infection (median of 7 [IQR 5-8] vs. 3 [IQR 2-4], p=0.003) (Figures 2, 3).

**Figure 2:**
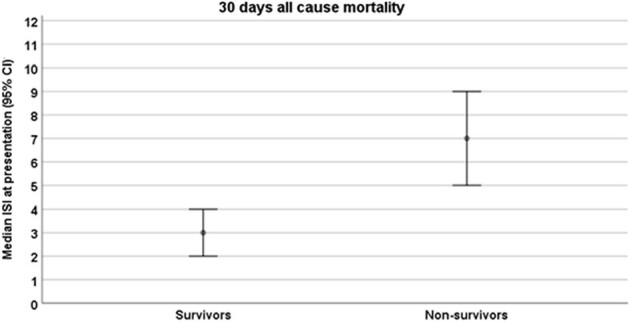
Median immunodeficiency scoring index at presentation according to 30-day mortality status (among patients with RVI, n=40). Abbreviations: ISI= immunodeficiency scoring index, RVI= respiratory virus infection.

**Conclusion:**

The ISI was associated with severe respiratory infections and 30-day mortality in a cohort of HCT recipients with different RVIs. The ISI can potentially be used to predict poor clinical outcomes in HCT recipients infected with any RVI and allow for early management of high-risk patients.

**Figure 3:**
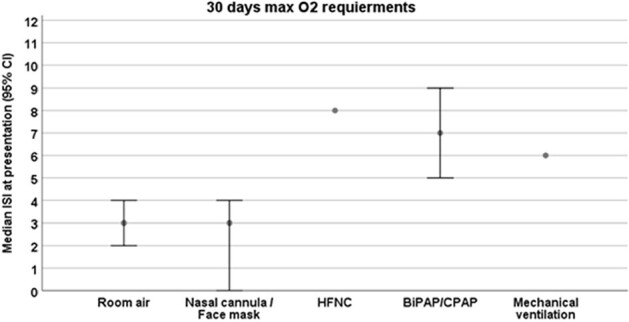
Median immunodeficiency scoring index at presentation according to 30-day maximal oxygen requirements (among patients with RVI, n=40). Abbreviations: BiPAP= bilevel positive airway pressure, CPAP= continuous positive airway pressure, HFNC= high flow nasal cannula, ISI= immunodeficiency scoring index, RVI= respiratory virus infection.

**Disclosures:**

**Fareed Khawaja, MBBS**, Eurofins Viracor: Grant/Research Support|Symbio: Grant/Research Support **Roy F. Chemaly, MD/MPH**, AiCuris: Advisor/Consultant|AiCuris: Grant/Research Support|Ansun Pharmaceuticals: Advisor/Consultant|Ansun Pharmaceuticals: Grant/Research Support|Astellas: Advisor/Consultant|Eurofins-Viracor: Grant/Research Support|InflaRX: Advisor/Consultant|Janssen: Advisor/Consultant|Karius: Advisor/Consultant|Karius: Grant/Research Support|Merck/MSD: Advisor/Consultant|Merck/MSD: Grant/Research Support|Moderna: Advisor/Consultant|Oxford Immunotec: Advisor/Consultant|Oxford Immunotec: Grant/Research Support|Roche/Genentech: Advisor/Consultant|Roche/Genentech: Grant/Research Support|Shinogi: Advisor/Consultant|Takeda: Advisor/Consultant|Takeda: Grant/Research Support|Tether: Advisor/Consultant

